# Mortality rate and predictors among stroke patients in the public hospitals in Harari region, Eastern Ethiopia

**DOI:** 10.1371/journal.pgph.0004414

**Published:** 2025-09-05

**Authors:** Alemayehu Tesfaye, Lemma Demissie Regassa, Birhanu Shegene, Nano Belema Areda, Assefa Tola

**Affiliations:** 1 School of Public Health, College of Health and Medical Sciences, Haramaya University, Harar, Ethiopia; 2 Department of Midwifery, College of Medicine and Health Science, Dire Dawa University, Dire Dawa, Ethiopia; Kwame Nkrumah University of Science and Technology, GHANA

## Abstract

Stroke is a major cause of death and disability worldwide, yet there is limited information on the mortality rate and its predictors in Eastern Ethiopia. This lack of evidence is particularly significant, as hospitals in the Harari region provide the majority of healthcare services for stroke and chronic diseases. Therefore, our objective is to assess the mortality rate and predictors among stroke patients in the public hospitals in Harari. An institutional-based retrospective follow-up study was conducted among 452 randomly selected stroke patients at public hospitals in the Harari region from July 1, 2019, to June 30, 2024. The incidence of unfavorable treatment outcomes was calculated at 95% CI, and predictors of mortality were determined using Cox regression analyses. Of the 452 patients included, 292 (64.6%) improved, 21 (4.7%) were discharged with complications, 63 (13.9%) died, and 76 (16.8%) were discharged against medical advice. The 60-month follow-up revealed a mortality rate of 7.6 (95% CI: 5.9–9.7) per 1,000 person-months. The mortality risk was higher among stroke patients with hypertension (AHR: 2.0, 95% CI: 1.1-3.9), heart failure (AHR: 2.2, 95% CI: 1.1, 4.9), those with complications (AHR: 4.9, 95% CI: 1.5, 16.3), hospital-acquired infections (AHR: 3.1, 95% CI: 1.5-6.7), aspiration pneumonia (AHR: 1.9, 95% CI: 1.1-3.4), poor Glasgow Coma Scale (GCS) scores (AHR: 6.9, 95% CI: 2.4-19.9), and moderate impairment in GCS (AHR: 4.7, 95% CI: 1.6-13.3). Conversely, the use of antiplatelet drugs was associated with a reduced mortality risk in stroke patients (AHR: 0.5, 95% CI: 0.3-0.9). The mortality rate of stroke in this study was comparable to that of other studies in Ethiopia. Factors such as hypertension, heart failure, lower GCS, complications, aspiration pneumonia, and hospital-acquired infections increased mortality risks, while antiplatelet drugs reduced them. Therefore, strategies for early screening and follow-up of at-risk patients are essential.

## Introduction

Stroke is a serious public health concern that can occur suddenly and result in significant mortality and disability. It accounts for 11.6% of all mortality and 57% of Disability-Adjusted Life Years (DALYs), making it an important cause of mortality and disability globally. Between 1990 and 2019, there was a 43% rise in stroke-related mortality and a 32% increase in stroke-related DALYs [1]. Stroke is responsible for five to six million deaths globally each year; on average, one stroke-related mortality occurs every four minutes [2]. For patients with ischemic strokes, administering hypolipidemic and antiplatelet medications is associated with improved outcomes. Likewise, those experiencing hemorrhagic strokes tend to have better results when treated with hypolipidemic and antihypertensive medications [3]. Sixty percent of high-income countries (HICs) and 26% of nations with low and middle incomes offered acute stroke treatments [4]. Around 70% of mortality due to stroke and 87% of stroke-related disabilities happen in nations with low and middle incomes [5]. It’s noteworthy that case fatalities appear to be rising in low and middle-income countries (LMICs) as opposed to HICs, underscoring the need for improved stroke care in LMICs [6]. Stroke inpatient mortality was high in sub-Saharan Africa. Stroke-related mortality was estimated to be 22% of the total population. In comparison to Eastern Africa (15%) and Southern Africa (18%), Western Africa had a higher stroke mortality rate (37%) [7].

Stroke fatalities among West Africans are linked to six patient characteristics: poor vegetable intake, elevated arterial pressure at presentation, more significant tumor volumes, elevated intracranial pressure (ICP), severe stroke, and aspiration pneumonia [8]. Population awareness, more intensive care unit (ICU) beds, telehealth, monitoring for late complications, and primary prevention, on the other hand, can lower in-hospital mortality rates [9]. Several conditions predicting mortality following an acute stroke were age, stroke type, stroke location, Glasgow Coma Scale degree of consciousness, NIHSS stroke severity, and comorbidities [9]. Stroke is associated with an increased risk of post-stroke infections [10], and they are a severe consequence for stroke patients in underdeveloped countries with insufficient rehabilitation services [11].

In Ethiopia, the severity of stroke and the need for better care are highlighted by the fact that almost one-fifth of stroke patients passed away while in the hospital [12]. The results of stroke treatment vary by region and over time. Stroke accounted for 7.5% to 19.3% of hospital admissions and 11% to 42.8% of fatalities from 2014 to 2019 [13]. Stroke-related complications were important indicators of death in Ethiopia [14].

Furthermore, stroke patients in Ethiopia typically have a dismal prognosis. Due to a shortage of computed tomography, excessively long prehospital delays, and a lack of essential drugs like r-tPA, most stroke patients experienced both neurologic and medical problems [15]. Data on stroke mortality are vital for tracking disease trends and organizing public health initiatives. Stroke mortality is a significant outcome metric in clinical trials and studies on stroke epidemiology. Effective management of adult stroke patients requires the identification of mortality predictors. Yet, information regarding treatment outcomes and mortality predictors is insufficient in Harari Regional State. Therefore, assessing mortality rate and its predictions is the aim of the current study.

## Method and material

### Ethics statement

The study was carried out under consideration of the Helsinki Declaration of Medical Research Ethics [16]. This work has been approved by the Institutional Health Research Ethical Review Committee of the Haramaya University College of Health and Medical Sciences (Ref. No.. IHRERC/175/2024). Permission was obtained from the Haramaya University College of Health and Medical Science administration. The names of patients were not registered in the checklist, and their unique MRN numbers were locked for confidentiality. The need for written informed consent to participate was waived by the Institutional Health Research Ethical Review Committee of the Haramaya University College of Health and Medical Sciences due to the retrospective nature of the study. Data were accessed from July 15 to August 1, 2024.

### Study setting and design

An institutional-based retrospective follow-up study design was conducted among stroke patients at public hospitals in the Harari region, 526 km away from Ethiopia’s capital city, Addis Ababa, from July 15 to August 1, 2024. There are currently two public, two private, one police, and one non-government hospital serving these individuals. In addition to the hospitals, the region’s population is served by nine health facilities, twenty-nine private clinics, twenty-six health posts, and one regional laboratory. Jugal General Hospital (JGH) and Hiwot Fana Comprehensive Specialized University Hospital (HFCSUH) are public hospitals in the Harari area that offer medical care to residents. Surgery, internal medicine, neurology services, mental health care, gynecology and obstetrics, pediatrics, maternal and child health (MCH), dental care, ophthalmology, TB and HIV (TB/HIV), intensive medical care, dermatology, and venereal disease services, pharmacy, oncologic services, and laboratory services are among health services they provide to the community [17].

### Populations and eligibility

All patients with stroke who were treated at public hospitals in Harari Regional State and whose age is greater than or equal to 15 years were the source population, whereas all stroke patients who were registered and admitted at the adult medical ward of public hospitals in the Harari region between July 1, 2019, and June 30, 2024, were our study population. All stroke patients greater than or equal to 15 years who were admitted to the medical ward in the study period were selected randomly and included in the study, whereas stroke patients with incomplete medical records about treatment outcome and an unknown date of diagnosis were excluded from the study.

### Sampling methods

The sample size for the first objective was calculated using the single population proportion formula by considering a similar study [18] with a 5% margin of error and a 95% confidence level. The sample size for the second objective (predictors of mortality of stroke patients) was calculated by considering the double population proportion formula and different factors that were significantly associated with the outcome variable, like types of strokes (hemorrhagic stroke) with assumed effect size AHR = 2.03 [19], Aspiration pneumonia AHR = 6.57 [20], Atrial fibrillation AHR = 1.104 [21], a two-sided confidence level of 95%, a margin of error of 5%, and a power of 80%, using EPI Info version 7 StatCalc software.

After comparing the results of the calculated sample size, a larger sample size of 434 was taken for this study. By adding a contingency of 10%, the final minimum sample size was 477 patients with stroke. A sampling frame was constructed by registering medical record numbers from the logbook of stroke patients. A total of 655 stroke patients (590 from HFCSUH and 65 from Jugal General Hospital) were registered and treated between January 1, 2019, and June 30, 2024. Based on the number of stroke patients at each Hospital, a proportionate amount of a 477-person sample was assigned to each. Finally, the required number of participants was selected by simple random sampling using a computer-generated random sample from the sampling frame (430 patients were randomly selected from HFCSUH, and 47 patients were randomly selected from Jugal General Hospital).

### Data collection instrument and procedure

The data was collected using a data extraction format that was adapted from the WHO STEPSwise approach to stroke surveillance [22]. Data abstraction formats that contained relevant information about the patients, like demographic characteristics, outcomes of treatment, comorbidity, medication used for treatment, laboratory investigations, and clinical data, were used to abstract the data. Data were collected by four public health staff members of Haramaya University, and data collection was supervised by two trained supervisors. A patient’s medical records (paper) were used to collect demographic characteristics, clinical data such as vital signs and types of strokes, comorbidity, medication for treatment, and outcomes of treatment.

### Variables

#### Dependent variable.

In-hospital mortality.

#### Independent variables.

Demographic factors (age, sex, residency)

Clinical-related factors (previous stroke, types of strokes, hypertension, DM, atrial fibrillation, aspiration pneumonia, raised intracranial pressure, Heart failure, kidney injury, dyslipidemia)

Treatment-related factors (lack of CT scan, prescribed medication, and the median time from onset of symptom to hospitalization).

### Operational definitions

#### Treatment outcomes.

The results or effects of medical intervention on a patient’s health (improved, complications, or death) [3].

#### Improved.

Information about the improvement of patients was obtained from the discharge summary of medical records. Functional outcome was evaluated by the modified Rankin Scale (mRS). It was categorized into good (mRS < 3) and poor (mRS ≥ 3) functional recovery [23].

#### Unfavorable treatment outcomes.

Negative results or effects that occur as a consequence of medical treatment [24]. These can include death, discharge with complications, and discharge against medical advice in this study.

#### Good GCS.

Refers to a patient with a mild brain injury or who is alert (GCS 13–15); a moderate brain injury or who is drowsy (GCS 9–12); poor GCS is a patient with severe brain injury or who is unconscious (GCS 3–8) [25].

#### Heart failure.

Diagnosed through a combination of patient history, physical examination, plus imaging- echocardiograms.

#### Event.

A stroke patient who passed away from any cause while receiving treatment [13].

#### Time to death.

Time from the date of stroke diagnosis to the date of death [13].

#### Survival status.

The status of the patients’ survival to the outcome (death) or censored [13].

### Data quality control

A pre-test was done in both hospitals for 5% of hospitalized stroke patients’ medical records to ensure the reliability and variability of the data collection tools, and then all necessary adjustments were made to the data collection instruments. Data collectors were trained for a day on how to collect the data. Supervision was provided by the principal investigators during the data collection process, and any inconsistencies were amended on time.

### Methods of data analysis

The data collected by the Kobo tool was checked for consistency and completeness, sent to the server, imported, and analyzed by STATA software version 17.0. Descriptive statistics (mean, frequencies, tables, and graphs) were used to summarize and describe the data. A complete case analysis of a dataset with missing completely at random was used to deal with missing data. The cumulative incidence of mortality was calculated by taking the number of deaths as the numerator and the total initial population at risk on follow-up as the denominator. Patient-months at risk of mortality were calculated from the baseline diagnosis date to either the date of events or censoring. Accordingly, incidence density was computed as the number of deaths by patient-months at risk. The outcome variables were dichotomized into death (event) and censored. The Kaplan-Meier failure curve and a log-rank test were used to estimate the probability of mortality and to test the equality of failure functions among explanatory variables, respectively. Cox PH was fitted to identify the predictors of mortality. The Schoenfeld residuals test (both global and scaled) and graphical (log-log plot of survival) methods were used to check the proportional hazard (PH) assumption. The presence of multicollinearity was checked by using the variance inflation factor. Variables with a p-value of ≤0.2 were entered by stepwise regression into a multivariable Cox regression model to control for the possible effect of confounders. A P-value <0.05 was used to declare statistical significance in the multivariable model, and the hazard ratio (HR) with its 95% confidence interval will be computed to show the strength of the association.

## Results

### Socio-demographic and clinical characteristics of the Study Participants

**Fig 1:**
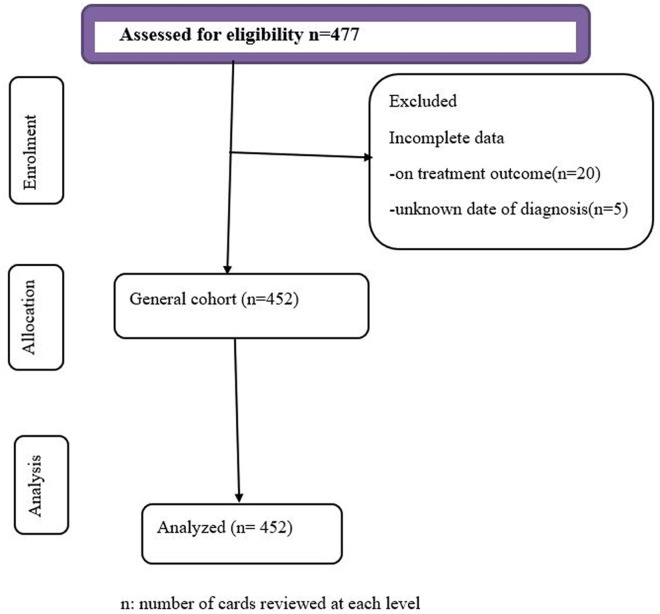
Flow diagram of selection for study on mortality rate and its predictors among stroke patients at public hospitals in the Harari region, East Ethiopia, 2024.

A total of 477 records were screened, and 25(5.2%) patients with incomplete information were excluded (Fig 1). Of the total 452 study participants, the majority (90.0%) were from Hiwotfana Hospital; about two-thirds of them were males, 190 (42.0%) had ischemic stroke, and the mean age (±SD) was 55 years (±15.6). The most common clinical presentation was hemiparesis (76.8%), followed by loss of consciousness (32.3%), slurred speech (25.0%), and headache (24.3%). The median time of presentation from symptom onset was 36 (IQR: 54.0) hours, and about 53.5% of the time, symptom onset to admission was > 24 hrs. The majority of them, 337 (74.6%), had comorbidity. Hypertension (55.3%) was the most common comorbid disease, followed by heart failure (11.7%), diabetes (11.1%), and kidney disease (9.3%). At hospitalization, the mean (SD) diastolic blood pressure and systolic blood pressure were 81.2 ± 18.7 mmHg and 137.5 ± 27.7 mmHg, respectively. About 8.2% of them had an elevated body temperature (>37.5°C). The mean total cholesterol was 145.4 ± 48.9 mg/dl, and 13.6% of the patients had elevated total cholesterol levels; the median high-density lipoprotein (HDL) was 42 ± 21.0. The median length of hospital stay was 5 ± 6.0 days (Table 1).

**Table 1:** Socio-demographic and clinical characteristics of stroke patients at public hospitals in the Harari region, East Ethiopia, from 2019 to 2024. (N = 452).

Characteristics	Category	Frequency (%)
Sex	Male	300(66.4)
	Female	152(33.6)
Age	<45	109 (24.1)
	45-65	225 (49.8)
	>65	118 (26.1)
Residence	Rural	299(66.2)
	Urban	153(33.9)
Type of stroke	Ischemic	190 (42.0)
	Hemorrhagic	160 (35.4)
	Unclassified	102 (22.6)
Clinical presentations	Hemiparesis	347 (76.8)
	Loss of consciousness	146 (32.3)
	Slurred speech	113 (35.0)
	Headache	110 (24.3)
	Aphasia	93 (20.6)
	Vomiting	67 (14.8)
	Facial palsy	62 (13.7)
Previous history of stroke	No	382 (84.5)
	Yes	70 (15.5)
Time from onset to admission (in hrs.)	Median ±(IQR) =36 ± 54.0	
Comorbidity	Yes	337 (74.6)
	No	115 (25.4)
List of comorbidities	Hypertension	250 (55.3)
	Heart failure	53 (11.7)
	Diabetes	50 (11.1)
	Kidney disease	42 (9.3)
	Myocardial Infraction	20 (4.4)
	Atrial fibrillation	16 (3.5)
Systolic blood pressure (in mmHg)	Mean**±**(SD)= 137.46 ± 27.7	
Diastolic blood pressure (in mmHg)	Mean**±**(SD)=81.24** ± **18.7	
Respiratory rate (in breaths per minute)	12-18	22 (4.9)
	>18	430 (95.1)
Pulse rate (in beats per minute)	<60	10 (2.2)
	60-100	353 (78.1)
	>100	89 (19.7)
Body temperature(in°C)	<36.5	192 (42.5)
	36.5-37.5	223 (49.3)
	>37.5	37 (8.2)
Random blood glucose (in mg/dl)	≤200	425 (94.0)
	>200	27 (6.0)
Total cholesterol (mg/dl)	<200	336 (86.4)
	≥200	53 (13.6)
Triglycerides(mg/dl)	<150	315 (81.4)
	≥150	72 (18.6)
HDL (mg/dl)	Median ±(IQR) **=**42 ± 21.0	
LDL (mg/dl)	Median ±(IQR) =55.7 ± 50.8	
Serum creatinine (in mg/dl)	<0.5	35 (7.7)
	0.5-1.2	354 (78.3)
	>1.2	63 (13.9)
Serum potassium level (in meq/l)	<3.5	84 (18.6)
	3.5-5	342 (75.7)
	>5	26 (5.7)
Glasgow Coma Scale (GCS)	·8	59 (13.1)
	9-12	152 (33.6)
	13-15	241 (53.3)
length of hospital stays(days)	Median ±(IQR) **=**5 ± 6.0	

### Treatment outcomes of stroke patients

From the total of 452 patients, 292 (64.6%) 95% CI (60.1%–68.9%) had improved outcomes, 21 (4.7%) 95% CI (3.0%–7.0%) were discharged with complications, about 63 (13.9%) 95% CI (11.0%–17.5%) died, and about 76 (16.8%) 95% CI (13.6%–20.6%) were discharged against medical advice on self and family request. About 160 (35.4%, 95% CI (31.1%–39.9%) had unfavorable outcomes. Of 63 patients who died during hospitalization, 31 (49.2%) had a hemorrhagic stroke. Aspiration pneumonia 37 (58.7%) and increased intracranial pressure 35 (55.6%) were the most frequently documented causes of death secondary to stroke. Of 160 unfavorable treatment outcomes that occurred during the follow-up period, more than half, 105 (65.6%), of them were males, and 83 (51.9%) were aged 45–65 years. Similarly, 111 (69.4%) had complications, and 94 (58.8%) were hypertensive at baseline. Among 452 patients, about 213 (47.1%) had complications. The most common complications during hospitalization were aspiration pneumonia (20.1%) and ICP/brain edema (20.4%). During hospitalization, the most commonly used antiplatelet and lipid-lowering drugs were Aspirin and Atorvastatin, administered to approximately 54.4% and 59.7% of the patients, respectively. Enalapril, which was administered in around 29.4% of cases, was the antihypertensive drug that stroke patients used the most frequently. Ceftriaxone and metronidazole were administered to 28.5% of hospitalized stroke patients to treat aspiration pneumonia and sepsis, as well as to control the effects of stroke and comorbidities (Table 2).

**Table 2:** Treatment outcomes for stroke patients at public hospitals in the Harari region, East Ethiopia, from 2019 to 2024. (N = 452).

Characteristics	Category		Frequency (%)
Treatment outcomes	Improved		292 (64.6)
	Unfavorable	Discharged with complications	21 (4.7)
		Died	63 (13.9)
		DAMA	76 (16.8)
Complication	Yes		213 (47.1)
	No		239 (52.9)
Lists of complications	Brain edema/ICP		92 (20.4)
	Aspiration pneumonia		91 (20.1)
	UTI		36 (8.0)
	HAI		32 (7.1)
	Seizure		21 (4.7)
	Septic shock		19 (4.2)
Anti-platelet	Aspirin		246 (54.4)
	Clopidogrel		22 (4.9)
Anticoagulant	Heparin		188 (41.6)
	Warfarin		32 (7.1)
Statins	Atorvastatin		270 (59.7)
Anti-hypertensive	Enalapril		133 (29.4)
	Amlodipine		116 (25.7)
	Nifedipine		56 (12.4)
	Hydrochlorothiazide		46 (10.2)
	Captopril		16 (3.5)
Anti-ulcer drugs	Cimetidine		69 (15.3)
	Omeprazole		19(4.2)
Antibiotics	Ceftriaxone		118 (26.1)
	Metronidazole		11(2.4)
Anti-pyretic	Paracetamol		119 (26.3)

### Overall survival rate of stroke patients

**Fig 2:**
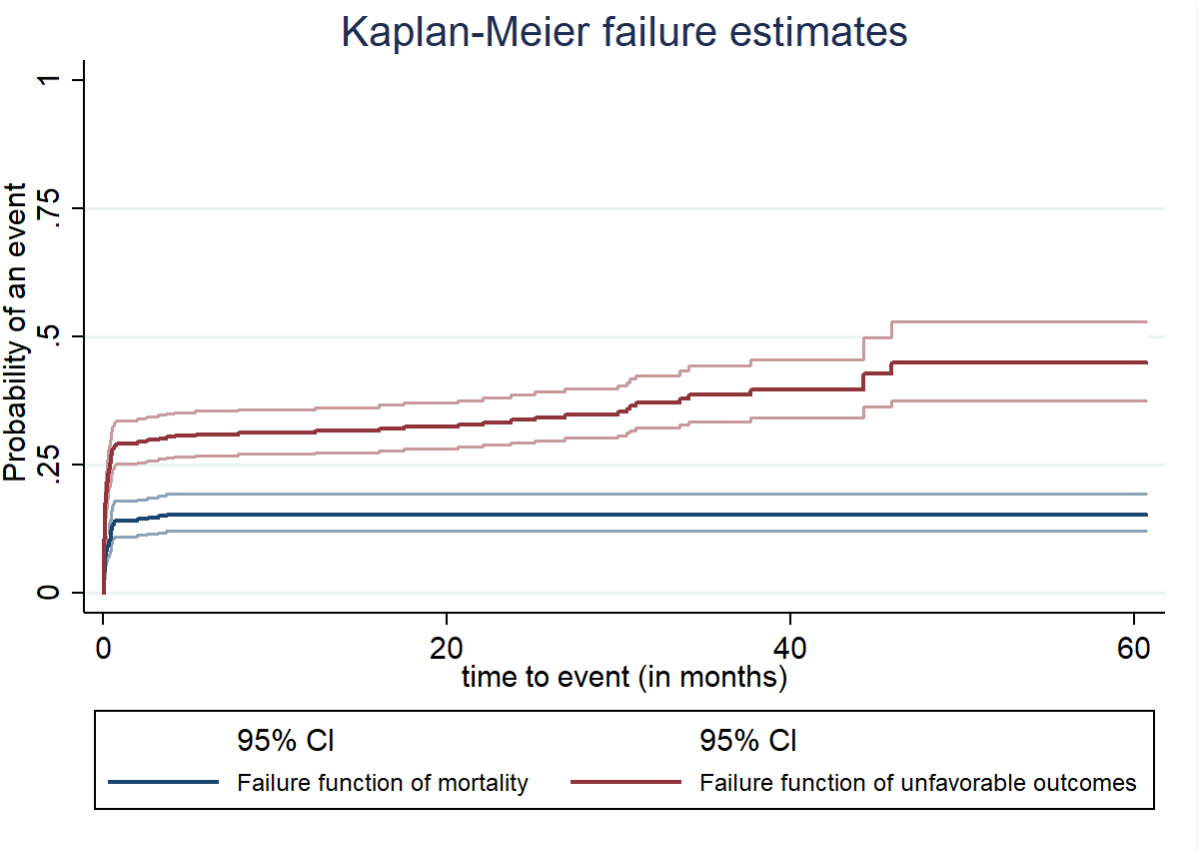
Overall Kaplan-Meier curve for the cumulative probability of mortality and unfavorable outcomes of stroke patients at public hospitals in the Harari region, East Ethiopia, 2024.

The participants were followed for a lowest of one day to a maximum of sixty months with no median survival time. Among the total stroke patients followed for approximately 60 months, 63 (13.9%, 95% CI (11.0%–17.5%) died, and 160 (35.4%, 95% CI (31.1%–39.9%) had unfavorable outcomes. The incidence of death was 7.6 cases per 1,000 person-months of observation with a 95% CI of (5.9–9.7). The cumulative probability of death among patients with stroke on the first day of admission was 2.5%; in the first month, it was 14.2%; in the third month, it was 14.7%; and from the fourth month to the end of the follow-up period, it was 15.4%. The incidence of unfavorable outcomes was 22.7 per 1,000 person-months of observation with a 95% CI of (19.4–26.5). The cumulative probability of unfavorable outcomes among patients with stroke on the first day of admission was 5.1%; at 20.2 months, it was 33.1%; at 40.2 months, it was 40.3%; and at the end of the follow-up period, it was 45.4% (Fig 2).

**Fig 3:**
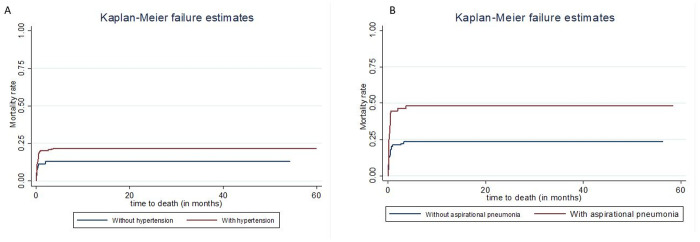
Kaplan-Meier curve for mortality estimate among different groups of stroke patients by hypertension (A) and aspiration pneumonia (B) at public hospitals of Harari region, East Ethiopia, 2024.

Kaplan Meier failure curve and a log-rank test showed that the differences in cumulative probability of death of patients with hypertension and those without hypertension, with aspiration pneumonia and those without aspiration pneumonia were statistically significant (log‐rank: **p* *= 0.0017, log‐rank: **p* *= 0.0001, respectively) (Fig 3).

### Predictors of mortality

In the bi-variable Cox regression analysis, ten variables (age greater than 65 years, hypertension, heart failure, lower GCS, having complications, aspiration pneumonia, increased intracranial pressure, hospital-acquired infection, respiratory rate > 18, and antiplatelet drug) were identified as the associated factors of mortality at p · 0.25. In the multivariable Cox regression analysis, seven variables were identified as predictors of mortality. These were hypertension, heart failure, lower GCS, having complications, aspiration pneumonia, hospital-acquired infection, and antiplatelet drugs.

Accordingly, hypertensive patients had a 2-fold higher probability of death because of stroke than non-hypertensive patients (AHR: 2.0, 95% CI: 1.1, 3.9). The risk of mortality of stroke was 2.2 times higher among patients with heart failure as compared to those without heart failure (AHR: 2.2, 95% CI: 1.1, 4.9). Having acute complications increased the hazard of death by 4.9 times compared to stroke patients who did not have complications (AHR: 4.9, 95% CI: 1.5, 16.3). The hazard of death from stroke was 3.1 times higher among patients with hospital-acquired infection as compared to those without hospital-acquired infection (AHR: 3.1, 95% CI: 1.5, 6.7). Stroke patients with aspiration pneumonia were 1.9 times more likely to die as compared to those without aspiration pneumonia (AHR: 1.9, 95% CI: 1.1, 3.4). The risk of mortality of stroke was 6.9 times higher among patients with GCS < 8 as compared to those with GCS 13–15 (AHR: 6.9, 95% CI: 2.4, 19.9). The risk of experiencing mortality from stroke was 4.7 times higher among patients with GCS 9–12 as compared to those with GCS 13–15 (AHR: 4.7, 95% CI: 1.6, 13.3). Furthermore, the risk of mortality was 50.0% lower among stroke patients who took antiplatelet drugs as compared to those who didn’t take antiplatelet drugs (AHR: 0.5, 95% CI: 0.3, 0.9) (Table 3).

**Table 3:** Bivariate and multivariate Cox regression analysis of predictors of mortality among stroke patients at public hospitals in the Harari region, East Ethiopia, 2024.

Variable	Category	Mortality		*CHR (95% CI)*	*AHR (95% CI)*	*P-value*
		*Censored(n = 389)*	*Event (n = 63)*			
Age	<45	99	10	1	1	
	45-65	202	23	0.9(0.5- 2.0)	1.1(0.4-2.4)	0.943
	>65	88	30	3.0(1.4-6.1)	1.6(0.7-3.6)	0.232
Residence	Rural	254	45	1.2(0.7-2.1)	1.1(0.6-1.9)	0.948
	Urban	135	18	1	1	
Hypertension	No	188	14	1	1	
	Yes	201	49	2.5(1.4- 4.6)	2.0(1.1- 3.9)	**0.033**
Heart Failure	No	346	53	1	1	
	Yes	43	10	1.7(0.8-3.3)	2.2(1.1-4.9)	**0.045**
GCS	*·*8	24	35	28.7(11.1-73.8)	6.9(2.4-19.9)	**0.001**
	9-12	129	23	8.2(3.1-21.7)	4.7(1.6-13.3)	**0.004**
	13-15	236	5	1	1	
Complication	No	238	4	1	1	
	Yes	151	59	19.1(6.9-52.8)	4.9(1.5-16.3)	**0.009**
Aspiration	No	335	26	1	1	
Pneumonia	Yes	54	37	7.0(4.2-11.6)	1.9(1.1- 3.4)	**0.038**
Increased ICP	No	332	28	1	1	
	Yes	57	35	5.1(3.1-8.5)	1.3(0.7-2.4)	0.484
HAI	No	370	50	1	1	
	Yes	19	13	5.6(3.0-10.3)	3.1(1.5-6.7)	**0.003**
Respiratory rate (breaths per minute)	12-18	18	4	1	1	
	>18	371	59	1.1(0.3-2.6)	1.5(0.4- 5.4)	0.562
Antiplatelet	No	162	44	1	1	
	Yes	227	19	0.3(0.2-0.6)	0.5(0.3-0.9)	**0.014**

## Discussion

This study included 452 patients with stroke (42.0%), 95% CI (37.6%–46.7%), who were diagnosed with ischemic stroke (35.4%), 95% CI (31.1%–39.9%), had hemorrhagic stroke, and (22.6%) 95% CI (19.0%–26.7%) patients with unclassified. Thirty-one patients (19.4%) with hemorrhagic stroke died, 18 patients with ischemic stroke (9.5%), and 14 patients with unclassified stroke (13.7%). The findings of this study showed that 63 (13.9%, 95% CI (11.0%–17.5%) of the stroke patients died, with an incidence density of 7.6 per 1,000 person-months (PM) of observation.

This study’s cumulative incidence of mortality was in line with the study in Felege Hiwot Hospital, Ethiopia, 15.2% [13]; in North West Ethiopia, 14.5% [26]; in Ayder Comprehensive Specialized Hospital, Ethiopia, 14.9% [27]; in Debre Markos Comprehensive Specialized Hospital, Ethiopia, 12.8% [19]; in Gondar University Hospital, Ethiopia, 12.5% [28]. However, it was higher as compared to the study done by [29] at a comprehensive stroke care center in Kerala, India, 3.4%. On the other hand, the cumulative incidence in this study was lower than the study conducted in Sierra Leone (34.8%) [30], at University of Gondar Teaching Hospital, Tibebe Gion Comprehensive Specialized Hospital, and Felege Hiwot Referral Hospital, Ethiopia (27.1%) [31], at Mettu Karl Referral, Ethiopia (27.2%) [2], at Saint Paul’s Hospital Millennium Medical College, Ethiopia (30.7%) [32], at Jimma University Medical Center, Ethiopia (51.6%) [18].

This could be due to differences in sample size, follow-up period, types of strokes, complications, and comorbidities. In Sierra Leone, 178 patients were followed for a maximum of 12 months, whereas this study followed 452 patients for a maximum of 60 months. In a study at the University of Gondar, Tibebe Gion Comprehensive Specialized Hospital, and Felege Hiwot Referral Hospital, Ethiopia, 57.9% of participants had ischemic stroke, compared to 42.0% in this study. At St. Paul’s Hospital in Addis Abeba, 70.9% of 251 participants had hypertension, 43.8% had diabetes mellitus (DM), and 53.8% were hemorrhagic stroke patients. In contrast, 55.3% of 452 participants in this study had hypertension, 11.1% had DM, and 35.4% were hemorrhagic stroke patients. In Mettu, 56.9% of 202 participants had complications, while 52.9% of those in this study had no complications. In Jimma, 54% had ischemic stroke and 38% had DM, compared to 42.0% and 11.1%, respectively, in this study. The higher comorbidities and complications in other studies are associated with increased mortality. On the other hand, the Indian study used a prospective follow-up design.

Age older than 65 years was not statistically significant in the study. A related study conducted in North West Ethiopia found that stroke patients over the age of 65 had a 6.3 times higher risk of dying than those under the age of 45 [30]. This finding is congruent with several other studies, including those conducted in Beirut [33], Jimma University Medical Center, Ethiopia [34], and Felege Hiwot Hospital, Ethiopia [13]. It could be associated with aging, which makes blood walls less elastic and more vulnerable to injury. Fatty deposits may accumulate in the arteries as a result, increasing the risk of obstructions and stroke death [35]. Furthermore, other comorbid diseases that are linked to death are more common in older people [34].

Hypertension was positively associated with mortality in this study. Patients with hypertension were more likely to die from a stroke than those without the condition. According to research done in Sierra Leone [30], in Debre Markos, Ethiopia [19], at Jimma University Medical, Ethiopia [18], and at Ayder Comprehensive Specialized Hospital, Ethiopia [27]. Because hypertension raises the danger of blood clots that might block the passage of blood to the brain, it has a high stroke fatality rate. Additionally, it can harm and weaken blood vessel walls, increasing their vulnerability to rupture [36]. Uncontrolled hypertension might cause damage to other vital organs such as the heart and kidneys, which can further increase the risk of stroke mortality over time [37].

In stroke patients, aspirational pneumonia dramatically raised the risk of death. These results are consistent with research done in Sierra Leone [30], in Lusaka, Zambia [38], in Tanzania [39], at Tibebe Ghion and Felege Hiwot hospitals in Amhara, Ethiopia [40], and at Jimma University Medical Center, Ethiopia [41]. Aspirational pneumonia can lead to several respiratory problems, including acute respiratory distress syndrome and respiratory failure, which can worsen the course of treatment for stroke patients and raise their chance of dying [42].

Lower GCS was significantly associated with mortality among stroke patients. The Glasgow Coma Scale for moderate impairment [9–12] (AHR = 2.2) and severe impairment [3–8] (AHR = 2.4) were found to be statistically significant predictors of in-hospital mortality in a study carried out at Debre Markos Comprehensive Specialized Hospital in Ethiopia [28]. This finding is also in line with the finding of a study conducted in Lusaka, Zambia [38], at Tibebe Ghion and Felege Hiwot hospitals in Amhara, Ethiopia [40], Jimma University Medical Center, Ethiopia [41], and at Saint Paul’s Hospital Millennium Medical College, Ethiopia [32]. A lower GCS indicates a more severe level of neurological impairment, which is often associated with more extensive brain damage that is directly interrelated with the severity of stroke and risk of mortality [43].

Patients with heart failure were more likely to die from a stroke than those without the condition. This finding is consistent with the findings of a study conducted in Tanzania [39] and at Tingandogo University Hospital in Burkina Faso [44]. This may be due to the elevated risk of mortality from cardiovascular disease, which in turn increases the risk of mortality from cerebrovascular disease. Due to the reduced cardiac output, reduced blood flow to the brain, and additional side effects, such as pulmonary edema, heart failure, it carries a high risk of death [33].

Stroke patients who experienced complications had a greater chance of dying than those who did not. According to research conducted at the Felege Hiwot Referral Hospital, Tibebe Gion Comprehensive Specialized Hospital, and University of Gondar Teaching Hospital, the absence of problems at admission was found to be a factor affecting the 28-day death rate [31]. Complications are indicative of a greater level of physiological disruption and impaired organ function, which might result in adverse consequences [40].

The incidence of mortality due to stroke was greater among individuals with hospital-acquired infections in comparison to those without such infections. This observation corresponds with the findings of a multicenter prospective cohort study involving Lebanese stroke patients, which indicated that the occurrence of infectious complications served as a predictor of mortality at 1 month (HR = 4.2, p = 0.013) and overall mortality (HR = 3.0, p = 0.007, respectively) [45]. Stroke has the potential to impair the patient’s immune system, rendering them more susceptible to infections [46]. Hospital-acquired infections may contribute to the onset of sepsis, a critical and potentially fatal condition [47].

Furthermore, the risk of mortality was 50.0% lower among stroke patients who utilized anti-platelet medications in comparison to those who did not utilize anti-platelet medications. Antiplatelet medications such as aspirin or clopidogrel hinder the development of blood clots [48]. Antiplatelet therapy may decrease the extent of brain damage caused by ischemia and simultaneously diminish the probability of early recurrent ischemic stroke, thereby lowering the likelihood of early death and enhancing treatment outcomes [49]. Antiplatelet medications have been demonstrated to possess cardioprotective properties, diminishing the risk of heart failure and other cardiovascular issues that can lead to mortality in stroke patients [50].

Prehospital care-seeking delays are common among patients with stroke, especially in low-income countries. In this study, the average onset-to-door time is 36 hours. Patients who live farther from hospitals, have a lower level of education, diabetes, hyperlipidemia, or a history of stroke are more likely to experience delays [51]. According to a study conducted at Yekatit-12 Hospital Medical College, Ethiopia, age, place of residence, health insurance, and stroke onset time significantly influence the timeliness of seeking medical care [52].

### Strengths and limitations of the study

This study has clarified the current situation of unfavorable treatment outcomes and predictors of mortality among patients with stroke at public hospitals in the Harari region of East Ethiopia. Conducted over a 60-month follow-up period, it effectively highlights the long-term impacts of stroke treatment. The research was carried out at two sites with an adequate sample size, enabling a reflection of the regional burden of stroke and supporting the potential for generalizations. However, since the study is retrospective, which had problems with being unfinished/incomplete, even losing patient medical information, and some important factors that might have a significant association with stroke mortality (road conditions, distances between medical centers, public awareness, occupation, substance use, physical exercise, and educational status) could not be found on the medical cards and were not assessed. This could underestimate the findings and reduce the statistical power of the study. The study only included public hospitals, and 22.6% of cases are unclassified, which could introduce a bias. Furthermore, the study may have overestimated the rate of mortality due to stroke by assuming that stroke was the exclusive cause of all cases of mortality.

## Conclusion

The mortality rate of stroke in this study was comparable to that of other studies in Ethiopia. Having hypertension, heart failure, a lower GCS, having complications, aspiration pneumonia, and hospital-acquired infection increased the hazards of mortality among stroke patients, whereas taking antiplatelet drugs reduced the hazards of mortality among stroke patients.

Based on the study’s findings, we recommend that health professionals prioritize the care of stroke patients with hypertension, heart failure, lower Glasgow Coma Scale scores, and complications such as aspiration pneumonia and hospital-acquired infections. Administrators at HFCSH and JGH should ensure adherence to secondary prevention strategies, including the use of antiplatelet and anticoagulant medications, as well as treatments for managing risk factors like hypertension. Additionally, researchers are encouraged to conduct further prospective follow-up studies to evaluate the incidence and potential predictors of mortality among stroke patients.

## Supporting information

S1 Data This is the data set of a study of mortality rate and predictors among stroke patients. (XLSX)

## References

[1] Feigin VL, Brainin M, Norrving B, Martins S, Sacco RL, Hacke W. World Stroke Organization (WSO): Global Stroke Fact Sheet 2022. Int J Stroke. 2022;17(1):18–29. 34986727 10.1177/17474930211065917

[2] Ababu DG, Getahun AM. Determinants of Stroke Mortality through Survival Models: The Case of Mettu Karl Referral Hospital, Mettu, Ethiopia. Stroke Res Treat. 2022;2022:9985127. doi: 10.1155/2022/9985127 35186250 PMC8856786

[3] Daswani R, Mishra DN, Phate SD. Study of drug utilization and outcomes in stroke patients in a tertiary care hospital. JMPAS. 2021.

[4] Owolabi MO, Thrift AG, Martins S, Johnson W, Pandian J, Abd-Allah F, et al. The state of stroke services across the globe: Report of World Stroke Organization-World Health Organization surveys. Int J Stroke. 2021;16(8):889–901. doi: 10.1177/17474930211019568 33988062 PMC8800855

[5] Akinyemi RO, Ovbiagele B, Adeniji OA, Sarfo FS, Abd-Allah F, Adoukonou T, et al. Stroke in Africa: profile, progress, prospects and priorities. Nat Rev Neurol. 2021;17(10):634–56. doi: 10.1038/s41582-021-00542-4 34526674 PMC8441961

[6] Thayabaranathan T, Kim J, Cadilhac DA, Thrift AG, Donnan GA, Howard G. Global stroke statistics 2022. Int J Stroke. 2022;17(9):946–56. 35975986 10.1177/17474930221123175 PMC9980380

[7] Mohammed AS, Degu A, Woldekidan NA, Adem F, Edessa D. In-hospital mortality and its predictors among stroke patients in sub-Saharan Africa: A systemic review and meta-analysis. SAGE Open Med. 2021;9. doi: 10.1177/20503121211036789 34377477 PMC8326621

[8] Sarfo FS, Akpa OM, Ovbiagele B, Akpalu A, Wahab K, Obiako R, et al. Patient-level and system-level determinants of stroke fatality across 16 large hospitals in Ghana and Nigeria: a prospective cohort study. Lancet Glob Health. 2023;11(4):e575–85. doi: 10.1016/S2214-109X(23)00038-4 36805867 PMC10080070

[9] Vozniuk IA, Morozova EM, Prokhorova MV. Changes in the in-hospital mortality due to stroke and factors affecting its reduction in the European Union, Middle East, USA, Canada, Ethiopia and China. Annals of Clinical and Experimental Neurology. 2021;15(1). doi: 10.25692/acen.2021.1.2

[10] Elkind MSV, Carty CL, O’Meara ES, Lumley T, Lefkowitz D, Kronmal RA, et al. Hospitalization for infection and risk of acute ischemic stroke: the Cardiovascular Health Study. Stroke. 2011;42(7):1851–6. doi: 10.1161/STROKEAHA.110.608588 21546476 PMC3125478

[11] Donkor ES. Stroke in the 21st Century: A Snapshot of the Burden, Epidemiology, and Quality of Life. Stroke Res Treat. 2018;2018. doi: 10.1155/2018/3238165 30598741 PMC6288566

[12] Alene M, Assemie MA, Yismaw L, Ketema DB. Magnitude of risk factors and in-hospital mortality of stroke in Ethiopia: a systematic review and meta-analysis. BMC Neurol. 2020;20(1):309. doi: 10.1186/s12883-020-01870-6 32814556 PMC7437163

[13] Walelgn N, Abyu GY, Seyoum Y, Habtegiorgis SD, Birhanu MY. The Survival Status and Predictors of Mortality Among Stroke Patients at North West Ethiopia. Risk Manag Healthc Policy. 2021;14:2983–94. doi: 10.2147/RMHP.S322001 34285612 PMC8286726

[14] Adem F, Mohammed B, Nigussie S. In-hospital treatment outcomes of acute stroke and determinant factors in a teaching hospital in eastern Ethiopia. SAGE Open Med. 2023;11. doi: 10.1177/20503121221149537 36685794 PMC9846299

[15] Fekadu G, Chelkeba L, Melaku T, Gamachu B, Gebre M, Bekele F, et al. Management protocols and encountered complications among stroke patients admitted to stroke unit of Jimma university medical center, Southwest Ethiopia: Prospective observational study. Ann Med Surg (Lond). 2019;48:135–43. doi: 10.1016/j.amsu.2019.11.003 31788240 PMC6880120

[16] Shrestha B, Dunn L. The Declaration of Helsinki on Medical Research involving Human Subjects: A Review of Seventh Revision. J Nepal Health Res Counc. 2020;17(4):548–52. doi: 10.33314/jnhrc.v17i4.1042 32001865

[17] JGH H. Annual Report. Hiwot Fana Comprehensive Specialized Hospital and Jugal General Hospital. 2023.

[18] Negasa BW, Wotale TW, Lelisho ME, Debusho LK, Sisay K, Gezimu W. Modeling survival time to death among stroke patients at Jimma University Medical Center, Southwest Ethiopia: A retrospective cohort study. Stroke Research and Treatment. 2023;2023:1557133. doi: 10.1155/2023/1557133 38130889 PMC10733594

[19] Admas M, Teshome M, Petrucka P, Telayneh AT, Alamirew NM. In-hospital mortality and its predictors among adult stroke patients admitted in Debre Markos Comprehensive Specialized Hospital, Northwest Ethiopia. SAGE Open Med. 2022;10. doi: 10.1177/20503121221122465 36093420 PMC9459489

[20] Wubshet A, Fanta K, Gemachu TD, Birhanu A, Gudina EK. Clinical characteristics and short-term outcomes of adult stroke patients admitted to Jimma Medical Center, Ethiopia: a prospective cohort study. Pan Afr Med J. 2023;44:49. doi: 10.11604/pamj.2023.44.49.37588 37070028 PMC10105338

[21] Addisu ZD, Mega TA. Clinical Characteristics and Treatment Outcomes of Acute Ischemic Stroke with Atrial Fibrillation Among Patients Admitted to Tertiary Care Hospitals in Amhara Regional State: Retrospective-Cohort Study. Vasc Health Risk Manag. 2023;19:837–53. doi: 10.2147/VHRM.S447936 38145253 PMC10748565

[22] Goulart AC, Bustos IR, Abe IM, Pereira AC, Fedeli LM, Benseñor IM, et al. A stepwise approach to stroke surveillance in Brazil: the EMMA (Estudo de Mortalidade e Morbidade do Acidente Vascular Cerebral) study. Int J Stroke. 2010;5(4):284–9. doi: 10.1111/j.1747-4949.2010.00441.x 20636711

[23] Zewde Y, Alem A, Seeger SK. Magnitude and predictors of post-stroke cognitive impairment among Ethiopian stroke survivors: A facility-based cross-sectional study. Research Square. 2023. doi: rs-2852302

[24] Furie KL, Jayaraman MV. 2018 guidelines for the early management of patients with acute ischemic stroke. Am Heart Assoc. 2018;509–10. 10.1161/STROKEAHA.118.020176 29367335

[25] Zewudie AZ, Regasa T, Hambisa S, Nureye D, Mamo Y, Aferu T, et al. Treatment Outcome and Its Determinants among Patients Admitted to Stroke Unit of Jimma University Medical Center, Southwest Ethiopia. Stroke Res Treat. 2020;2020:8817948. doi: 10.1155/2020/8817948 33489080 PMC7790566

[26] Abebe TG, Feleke SF, Dessie AM, Anteneh RM, Anteneh ZA. Development and internal validation of a clinical risk score for in-hospital mortality after stroke: a single-centre retrospective cohort study in Northwest Ethiopia. BMJ Open. 2023;13(3):e063170. doi: 10.1136/bmjopen-2022-063170 36977538 PMC10069517

[27] Hagos Gufue Z, Gizaw NF, Ayele W, Yifru YM, Hailu NA, Welesemayat ET, et al. Survival of Stroke Patients According to Hypertension Status in Northern Ethiopia: Seven Years Retrospective Cohort Study. Vasc Health Risk Manag. 2020;16:389–401. doi: 10.2147/VHRM.S247667 33061400 PMC7533221

[28] Gebreyohannes EA, Bhagavathula AS, Abebe TB, Seid MA, Haile KT. In-Hospital Mortality among Ischemic Stroke Patients in Gondar University Hospital: A Retrospective Cohort Study. Stroke Res Treat. 2019;2019:7275063. doi: 10.1155/2019/7275063 30693082 PMC6332873

[29] Nambiar V, Raj M, Vasudevan D, Bhaskaran R, Sudevan R. One-year mortality after acute stroke: a prospective cohort study from a comprehensive stroke care centre, Kerala, India. BMJ Open. 2022;12(11):e061258. doi: 10.1136/bmjopen-2022-061258 36442894 PMC9710353

[30] Russell JBW, Charles E, Conteh V, Lisk DR. Risk factors, clinical outcomes and predictors of stroke mortality in Sierra Leoneans: A retrospective hospital cohort study. Ann Med Surg (Lond). 2020;60:293–300. doi: 10.1016/j.amsu.2020.10.060 33204420 PMC7649580

[31] Ayehu GW, Yitbarek GY, Jemere T, Chanie ES, Feleke DG, Abebaw S, et al. Case fatality rate and its determinants among admitted stroke patients in public referral hospitals, Northwest, Ethiopia: A prospective cohort study. PLoS One. 2022;17(9):e0273947. doi: 10.1371/journal.pone.0273947 36108071 PMC9477361

[32] Sahle Adeba T, Mekonen H, Alemu T, Alate T, Melis T. Survival status and predictor of mortality among adult stroke patients in Saint Paul’s hospital millennium medical college, Addis Ababa, Ethiopia. SAGE Open Med. 2022;10. doi: 10.1177/20503121221112483 35924142 PMC9340903

[33] Chen Z, Venkat P, Seyfried D, Chopp M, Yan T, Chen J. Brain–heart interaction: cardiac complications after stroke. Circulation Research. 2017;121(4):451–68. 28775014 10.1161/CIRCRESAHA.117.311170 PMC5553569

[34] Li T, Huang Y, Cai W, Chen X, Men X, Lu T, et al. Age-related cerebral small vessel disease and inflammaging. Cell Death Dis. 2020;11(10):932. doi: 10.1038/s41419-020-03137-x 33127878 PMC7603301

[35] Tyrrell DJ, Goldstein DR. Ageing and atherosclerosis: vascular intrinsic and extrinsic factors and potential role of IL-6. Nat Rev Cardiol. 2021;18(1):58–68. doi: 10.1038/s41569-020-0431-7 32918047 PMC7484613

[36] Meissner A. Hypertension and the Brain: A Risk Factor for More Than Heart Disease. Cerebrovasc Dis. 2016;42(3–4):255–62. doi: 10.1159/000446082 27173592

[37] Kjeldsen SE. Hypertension and cardiovascular risk: General aspects. Pharmacol Res. 2018;129:95–9. doi: 10.1016/j.phrs.2017.11.003 29127059

[38] Nutakki A, Saylor D, Chishimba L, Chomba M, Mataa M, Zimba S. Predictors of stroke mortality at university teaching hospital in Lusaka, Zambia. J Stroke and Cerebrovascular Diseases. 2022;31(4):106359. doi: 10.1016/j.jstrokecerebrovasdis.2022.106359

[39] Matuja SS, Mlay G, Kalokola F, Ngoya P, Shindika J, Andrew L, et al. Predictors of 30-day mortality among patients with stroke admitted at a tertiary teaching hospital in Northwestern Tanzania: A prospective cohort study. Front Neurol. 2023;13:1100477. doi: 10.3389/fneur.2022.1100477 36742055 PMC9889987

[40] Kortazar-Zubizarreta I, Pinedo-Brochado A, Azkune-Calle I, Aguirre-Larracoechea U, Gomez-Beldarrain M, Garcia-Monco JC. Predictors of in-hospital mortality after ischemic stroke: A prospective, single-center study. Health Sci Rep. 2019;2(4):e110. doi: 10.1002/hsr2.110 31049417 PMC6482326

[41] Mosisa W, Gezehagn Y, Kune G, Chego M, Yigezu HF, Getnet M. Survival status and predictors of mortality among adult stroke patients admitted to Jimma University Medical Center, South West Ethiopia: A retrospective cohort study. Vasc Health Risk Manag. 2023;19:527–41. 37649671 10.2147/VHRM.S399815 PMC10464890

[42] Feng M-C, Lin Y-C, Chang Y-H, Chen C-H, Chiang H-C, Huang L-C, et al. The Mortality and the Risk of Aspiration Pneumonia Related with Dysphagia in Stroke Patients. J Stroke Cerebrovasc Dis. 2019;28(5):1381–7. doi: 10.1016/j.jstrokecerebrovasdis.2019.02.011 30857927

[43] Ranasinghe VS, Pathirage M, Gawarammana IB. Predictors of in-hospital mortality in stroke patients. PLOS Glob Public Health. 2023;3(2):e0001278. doi: 10.1371/journal.pgph.0001278 36962904 PMC10021536

[44] Dabilgou AA, Dravé A, Kyelem JMA, Ouedraogo S, Napon C, Kaboré J. Frequency and Mortality Risk Factors of Acute Ischemic Stroke in Emergency Department in Burkina Faso. Stroke Res Treat. 2020;2020:9745206. doi: 10.1155/2020/9745206 32577197 PMC7305528

[45] Abdo R, Abboud H, Salameh P, El Hajj T, Hosseini H. Mortality and Predictors of Death Poststroke: Data from a Multicenter Prospective Cohort of Lebanese Stroke Patients. J Stroke Cerebrovasc Dis. 2019;28(4):859–68. doi: 10.1016/j.jstrokecerebrovasdis.2018.11.033 30638943

[46] Wang H, Zhang S, Xie L, Zhong Z, Yan F. Neuroinflammation and peripheral immunity: Focus on ischemic stroke. Int Immunopharmacol. 2023;120:110332. doi: 10.1016/j.intimp.2023.110332 37253316

[47] Blot S, Ruppé E, Harbarth S, Asehnoune K, Poulakou G, Luyt C-E, et al. Healthcare-associated infections in adult intensive care unit patients: Changes in epidemiology, diagnosis, prevention and contributions of new technologies. Intensive Crit Care Nurs. 2022;70:103227. doi: 10.1016/j.iccn.2022.103227 35249794 PMC8892223

[48] Kapil N, Datta YH, Alakbarova N, Bershad E, Selim M, Liebeskind DS, et al. Antiplatelet and Anticoagulant Therapies for Prevention of Ischemic Stroke. Clin Appl Thromb Hemost. 2017;23(4):301–18. doi: 10.1177/1076029616660762 27461564

[49] Minhas JS, Chithiramohan T, Wang X, Barnes SC, Clough RH, Kadicheeni M, et al. Oral antiplatelet therapy for acute ischaemic stroke. Cochrane Database Syst Rev. 2022;1(1):CD000029. doi: 10.1002/14651858.CD000029.pub4 35028933 PMC8758582

[50] Demirtas S, Karahan O, Yazıcı S, Guclu O, Calıskan A, Tezcan O, et al. Investigation of possible prophylactic, renoprotective, and cardioprotective effects of thromboprophylactic drugs against ischemia-reperfusion injury. Kaohsiung J Med Sci. 2015;31(3):115–22. doi: 10.1016/j.kjms.2014.12.005 25744233 PMC11916766

[51] Zhao Y, Xu Y, Fang S, Zhi S, Ma D, Song D, et al. Incidence and Associated Factors of Prehospital Care-Seeking Delay in People with Acute Ischemic Stroke: A Systematic Review and Meta-Analysis. Neuroepidemiology. 2024;1–16. doi: 10.1159/000542765 39701059

[52] Mamo BT, Tefera DB, Altaye MG, Geram FG, Dano AM, Sana YB, et al. Delay in hospital arrival and determinate factors among acute stroke Patients at Yekatit-12 Hospital Medical College, Ethiopia: Unmatched case-control study. 2024.

