# Supplementary material for: Mortality rate and predictors among stroke patients in the public hospitals in Harari region, Eastern Ethiopia
Source: PLOS Glob Public Health. 2025 Sep 5;5(9):e0004414. doi: 10.1371/journal.pgph.0004414 (PMC12412954; doi:10.1371/journal.pgph.0004414)
Supplement: Attachment — Submitted filename: Response to reviewers_stroke.docx [file pgph.0004414.s002.docx]

**Manuscript PGPH-D-25-00374**

**Response to reviewers**

Dear Dr. Joanna Tindall,

Thank you for giving us the opportunity to submit a revised draft of our manuscript titled “Mortality rate and predictors for stroke patients in the public hospital of Harari region, Eastern Ethiopia” to *PLOS Global Public Health*. We appreciate the time and effort that you and the reviewers dedicated to providing feedback on our manuscript and are grateful for the insightful comments and valuable improvements to our paper. We have now addressed the following concerns.

**Editor’s comments**

Journal Requirements:

1. "We noticed you have some minor occurrence of overlapping text with the following previous publication(s), which needs to be addressed:

<https://bmccardiovascdisord.biomedcentral.com/articles/10.1186/s12872-024-04106-4>

In your revision ensure you cite all your sources (including your own works), and quote or rephrase any duplicated text outside the methods section. Further consideration is dependent on these concerns being addressed."

**Author response:** Thank you. We addressed this concern accordingly. We went through the entire manuscript, and changes made are highlighted in red.

2. We ask that a manuscript source file is provided at Revision. Please upload your manuscript file as a .doc, .docx, .rtf or .tex.

**Author response:** Done.

3. In the online submission form, you indicated that Data upon which the result is based will be available from the corresponding author upon request.

All PLOS journals now require all data underlying the findings described in their manuscript to be freely available to other researchers, either

1. In a public repository,

2. Within the manuscript itself, or

3. Uploaded as supplementary information.

This policy applies to all data except where public deposition would breach compliance with the protocol approved by your research ethics board. If your data cannot be made publicly available for ethical or legal reasons (e.g., public availability would compromise patient privacy), please explain your reasons by return email and your exemption request will be escalated to the editor for approval. Your exemption request will be handled independently and will not hold up the peer review process, but will need to be resolved should your manuscript be accepted for publication. One of the Editorial team will then be in touch if there are any issues.

**Author response:** All data supporting the findings of this study are freely available as supplementary information.

Additional Editor Comments (if provided):

**Reviewer’s comments**

Reviewer #1: In their work, the authors present the epidemiology of stroke, treatment outcomes, and predictors of stroke-related mortality in Ethiopia.
The results of such a study may be useful in assessing the epidemiology of cerebrovascular diseases and in developing healthcare strategies in these regions.

**Author response**: Thank you!

However, the paper contains several shortcomings that should be addressed before publication in a journal.
Comments:
It is difficult to agree with the statement that "Although infections have received relatively little attention in stroke research" because the impact of infections on stroke risk and progression has been studied for many years. One of the first researchers in this area was Pierre Marie in 1885, followed by Zygmunt Freud, who—before becoming a distinguished psychiatrist and psychoanalyst—practiced as a neurologist in Vienna. In Freud’s research, infections preceded the onset of stroke in up to one-third of cases. Later, particularly in the 1990s, further studies were conducted on infections such as Helicobacter pylori and Chlamydia pneumoniae. See, for example, Mendall et al. (1965), Whinup et al. (1996), and Markus & Mendall (1998). More recently, additional studies have been published, such as in Cerebrovasc Dis Extra (2025 Mar 11:1-19) and Emsley HC, Hopkins SJ. "Acute Ischaemic Stroke and Infection: Recent and Emerging Concepts" (Lancet Neurol. 2008;7(4):341-53). Another relevant study is Elkind MS et al. "Hospitalization for infection and risk of acute ischemic stroke: the Cardiovascular Health Study" (Stroke. 2011;42(7):1851-6), which identifies infections as a risk factor for stroke.
In these studies, stroke was most commonly preceded by respiratory infections, which generally aligns with the authors' findings, although this knowledge is secondary to earlier research.

**Author response**: Thank you very much for pointing this out. We revised the sentence as follows:

“Infection is associated with a risk of stroke (13), and they are a severe consequence for stroke patients in underdeveloped countries with insufficient rehabilitation services (14) ”[Pg4, Ln11-13]

Next Comments:
• The observation period was quite long, spanning from July 1, 2019, to June 30, 2023. Unfortunately, the paper lacks clear information on the total number of patients admitted to all analyzed hospitals during this period. The authors only provide data on a pre-selected group of patients, which can introduce methodological bias, especially given the presence of numerous confounding factors that hinder an accurate assessment of the situation. However, based on the presented data, the authors had probably (? See above mentioned lack of clear information) access to well over 4,000 patients, which is a substantial number allowing for robust statistical conclusions.
• Maybe, it might have been better to conduct a longitudinal study with consecutive cases over a shorter period, including all patients? The authors should more thoroughly explain why they chose their particular study methodology.

**Author response**: Thanks for your comment. We have included the following paragraph in the methods section, Sampling methods subsection:

**“**A total of 655 stroke patients (590 from HFCSUH and 65 from Jugal General Hospital) were registered and treated between January 1, 2019, and June 30, 2024. Based on the number of stroke patients at each Hospital, a proportionate amount of 477-person sample was assigned to each. Finally, the required number of participants was selected by simple random sampling using a computer-generated random sample from the sampling frame (430 patients were randomly selected from HFCSUH, and 47 patients were randomly selected from Jugal General Hospital).” [Pg6, Ln5-11]

• Moreover, the time from symptom onset to hospital admission was notably long—36 hours. The authors should justify this delay, which is likely due to factors such as poor road conditions, long distances between medical centers, and low public awareness. However, it would be advisable for the authors to explicitly discuss these issues. On the other hand, such a long delay precludes effective acute-phase treatment.

**Author response**: Thank you very much for the comment. The time from symptom onset to hospital admission was not statistically significant in the study. We have specified the limitations in the discussion section, which need to be addressed by other scientists in the future.

Due to the retrospective nature of the study, some important factors (road conditions, distances between medical centers, public awareness, occupation, substance use, physical exercise, and educational status) could not be found on the medical cards and were not assessed [Pg14, Ln19-23].

• Additionally, the authors report that over 35% of patients had hemorrhagic stroke, which is a very high proportion although is known that studies have shown that individuals of African descent have a 2-3 times higher risk of experiencing a hemorrhagic stroke compared to the White population, it is also requires explanation, for readers not very familiar with stroke epidemiology

**Author response**: Thank you for this suggestion. Hypertension (55.3%) was the most common comorbid disease and the leading risk factor for hemorrhagic stroke, which occurred in 35.4% of the patients in our study. This finding is in line with the systematic review conducted in Ethiopia, which revealed that the pooled burden of hemorrhagic stroke was 46.42%. “The burden of stroke and modifiable risk factors in Ethiopia: A systematic review and meta-analysis” (Abate et al., 2021)
