# Supplementary material for: Mortality rate and predictors among stroke patients in the public hospitals in Harari region, Eastern Ethiopia
Source: PLOS Glob Public Health. 2025 Sep 5;5(9):e0004414. doi: 10.1371/journal.pgph.0004414 (PMC12412954; doi:10.1371/journal.pgph.0004414)
Supplement: Attachment — Submitted filename: Reviewer 2 Comments.docx [file pgph.0004414.s004.docx]

Reviewer 2 Comments

Title: The title of the manuscript needs to be modified.

• The title of the manuscript, ‘Mortality rate and predictors for stroke patients in the public hospital of Harari region, Eastern Ethiopia’, gives the impression that the authors are speaking of a single public hospital, which is the only public hospital in Harari. The authors should therefore consider modifying the title to ‘Mortality rate and predictors among stroke patients in public hospitals in the Harari region, Eastern Ethiopia’ or ‘Mortality rate and its predictors among stroke patients in public hospitals in the Harari region, Eastern Ethiopia’

Abstract

• Background: the background of the abstract neither reads well nor seems to convey the contextual significance that the authors appear to want to communicate. The authors need to rewrite to improve the clarity of thought and expression.

• Results: The authors need to clarify the criteria used to determine improvement in patients (e.g., functional outcome scale, NIHSS, or another metric). This should be included in the methods section of the main text.

• Kindly replace 'left against medical advice' with the more standard term 'discharged against medical advice.' Ensure consistency regarding the use of 'mortality rate' instead of 'death rate'.

• Conclusion: the conclusion ‘In-hospital mortality was observed in one of seven stroke patients treated at public hospitals.’ It is out of place and does not align with the results provided in the abstract.

Main text

• Introduction: The introduction should be succinct and straight to the point. The authors should concentrate on the scientific premise, evidence gap, healthcare environment, and regional burden that support the study. The definition of stroke, for instance, is unnecessary. The same applies to the paragraph on management options for stroke in a broader context rather than what pertains in their region. The first sentence of paragraph 3 of the introduction ‘The fact that 94% of ischemic stroke patients survived and 6% passed away is remarkable. Hemorrhagic stroke patients, on the other hand, had a survival rate of 88%, while 12% died (6).’ is out of place and should be deleted. I recommend that the authors make the introduction more concise.

Methods: The methods section requires a bit more detail.

• Populations and Eligibility: The exclusion criteria are very scanty. For example, were patients with incomplete medical records excluded? If so, this should be included as an exclusion criterion. How did you deal with missing data? Were the retrospective records retrieved from electronic health records, or paper charts, or were there existing stroke registries?

• What proportion of patients what neuroimaging confirmed strokes? The statement ‘whereas stroke patients whose primary diagnosis was not a stroke (e.g., transient ischemic attacks without stroke confirmation)’ is poorly structured and seems to suggest all patients had neuroimaging. Currently the diagnosis of transient ischemic attacks is tissue based and requires the use of neuroimaging to rule out strokes.

• The authors also state that ‘transfer-in patients within the follow-up period were excluded from the study’. Considering the hospitals being studied are tertiary facilities, I expect a significant proportion of the patients to be referred from lower-level facilities. Can the authors explain why they were excluded?

• Sampling methods. The authors need to clarify how the sampling frame was constructed and whether any pre-screening was done. How was the list of eligible patients compiled? Were exclusion criteria applied before this apparent random selection of participants?

• Also, it would be helpful to include a flow diagram indicating the total number of records screened. The proportion excluded and reasons for exclusion (the number excluded for incomplete records, transfer-in/ referred from other facilities, etc. and the final number of patients included.

• Regarding the sample size calculation for the second objective, specify assumed proportions or effect size. At least one key factor used.

• Variables: how was raised ICP? Was it via invasive ICP monitoring? For stroke patients, it is surprising to see heart failure contributing more than other comorbidities like diabetes. This is appropriately highlighted in the discussion section. It would be helpful to indicate how these heart failures were diagnosed in the methods, to clarify the possibility of overestimation. Did the patients have echocardiograms done? Also, why is dyslipidemia not included in the comorbidities?

• In retrospective chart reviews, it is impractical to say that "Authors had no access to information that could identify individual participants during or after data collection." This statement should be removed or revised.

Results:

• The authors state that there was a total of 477 samples which ambiguous. Reword to reflect the fact that a total of 477 records were screened. Also, screen failures should not be included in the total sample size. If patients with incomplete records were excluded, report this both in the methods and in the flow of the results.

• Further classify hemorrhagic stroke into subtypes (e.g., intracerebral hemorrhage, subarachnoid hemorrhage). Provide a more detailed breakdown of stroke-related complications.

• Do the authors have information regarding the length of stay of patients. Can this be incuded?

• There is no data included on the interventions received by patients such as thrombolysis, thrombectomy, EVD, aneurysmal clipping/ coiling etc. how did the treatments received by patients affect mortality?

• Kindly provide the appropriate labels for the Kaplan-Meier curve to enhance interpretability. The y-axis should be Labelled, and the x-axis should indicate follow-up time or time to event, not “analysis time.” Also, it would be helpful to stratify the curves by important factors such as aspiration pneumonia, etc.

Limitations:

• The study only included public hospitals, which could introduce significant bias. This should be included in the limitation.

• 22.6% of cases are unclassified. This is a significant limitation.

Response to previous reviewer comments

The authors have failed to adequately address some of the concerns raised by the previous authors. The authors are urged to see the review process as an endeavor to improve the manuscript's quality. Most of the recommendations from the previous reviewers should be incorporated to strengthen the paper.

1. The revision of the sentence ‘Although infections have received relatively little attention in stroke research’ to ‘Infection is associated with a risk of stroke’ remains out of context. A more accurate phrasing would be: ‘Stroke is associated with an increased risk of post-stroke infections, which can influence outcomes.

2. As was rightly pointed out by the previous reviewers, the average onset-to-door time of 36 hours is clinically significant and warrants discussion. . Although not a primary objective, this is a key finding and should be acknowledged and discussed using insights from relevant regional studies. The delay may reflect systemic issues such as poor road infrastructure, long distances to care, or low public awareness
