# Supplementary material for: Mortality rate and predictors among stroke patients in the public hospitals in Harari region, Eastern Ethiopia
Source: PLOS Glob Public Health. 2025 Sep 5;5(9):e0004414. doi: 10.1371/journal.pgph.0004414 (PMC12412954; doi:10.1371/journal.pgph.0004414)
Supplement: Attachment — Submitted filename: Response_to_reviewers2_stroke_auresp_3.docx [file pgph.0004414.s005.docx]

**Manuscript PGPH-D-25-00374R2**

**Response to reviewers**

Dear Dr. Elliot Koranteng Tannor,

Thank you for giving us the opportunity to submit a revised draft of our manuscript titled “Mortality rate and its predictors among stroke patients in the public hospital in Harari region, Eastern Ethiopia” to *PLOS Global Public Health*. We appreciate the time and effort that you and the reviewers dedicated to providing feedback on our manuscript and are grateful for the insightful comments and valuable improvements to our paper. We have now addressed the following concerns.

**Editor’s comments**

*Journal Requirements:*

*Please review your reference list to ensure that it is complete and correct. If you have cited papers that have been retracted, please include the rationale for doing so in the manuscript text, or remove these references and replace them with relevant current references. Any changes to the reference list should be mentioned in the rebuttal letter that accompanies your revised manuscript. If you need to cite a retracted article, indicate the article’s retracted status in the References list and also include a citation and full reference for the retraction notice*

**Author response:** Thank you. We revised the reference list accordingly, and it is complete and correct. We removed a few references to make the introduction concise and added additional references to specify the assumed effect size for key factors, as per the reviewer’s comments.

Additional Editor Comments (if provided):

**Reviewer’s comments**

*Reviewer #2: Title: The title of the manuscript needs to be modified.
• The title of the manuscript, ‘Mortality rate and predictors for stroke patients in the public hospital of Harari region, Eastern Ethiopia’, gives the impression that the authors are speaking of a single public hospital, which is the only public hospital in Harari. The authors should therefore consider modifying the title to ‘Mortality rate and predictors among stroke patients in public hospitals in the Harari region, Eastern Ethiopia’ or ‘Mortality rate and its predictors among stroke patients in public hospitals in the Harari region, Eastern Ethiopia’*

**Author response**: Thank you for pointing this out. We agree with this comment. Therefore, we have changed the title of the manuscript to ‘Mortality rate and its predictors among stroke patients in the public hospitals in Harari region, Eastern Ethiopia.’

*Abstract
• Background: the background of the abstract neither reads well nor seems to convey the contextual significance that the authors appear to want to communicate. The authors need to rewrite to improve the clarity of thought and expression*

**Author response**: Thank you very much. We have revised the paragraph as follows:

“Stroke is a major cause of death and disability worldwide, yet there is limited information on the mortality rate and its predictors in Eastern Ethiopia. This lack of evidence is particularly significant since hospitals in the Harari region provide most of the stroke and chronic disease healthcare services. Therefore, our objective is to assess the mortality rate and its predictors among stroke patients in the public hospitals in Harari.” [Pg2, Ln2-6]

• *Results: The authors need to clarify the criteria used to determine improvement in patients (e.g., functional outcome scale, NIHSS, or another metric). This should be included in the methods section of the main text.*

**Author response**: Thanks for your comment. We took the assessment directly from the patient’s medical records. We have included the following sentences in the methods section.

**“Improved**- Information about the improvement of patients was obtained from the discharge summary of medical records.” [Pg6, Ln22-23]

*• Kindly replace 'left against medical advice' with the more standard term 'discharged against medical advice.' Ensure consistency regarding the use of 'mortality rate' instead of 'death rate'.*

**Author response**: Done.

*• Conclusion: The conclusion, ‘In-hospital mortality was observed in one of seven stroke patients treated at public hospitals.’ It is out of place and does not align with the results provided in the abstract.*

**Author response**: Thank you for this suggestion. We have revised the sentences as follows:

“The mortality rate of stroke in this study was comparable to that of other studies in Ethiopia.” [Pg2, Ln21-22]

*Main text
• Introduction: The introduction should be succinct and straight to the point. The authors should concentrate on the scientific premise, evidence gap, healthcare environment, and regional burden that support the study. The definition of stroke, for instance, is unnecessary. The same applies to the paragraph on management options for stroke in a broader context rather than what pertains in their region. The first sentence of paragraph 3 of the introduction ‘The fact that 94% of ischemic stroke patients survived and 6% passed away is remarkable. Hemorrhagic stroke patients, on the other hand, had a survival rate of 88%, while 12% died (6).’ is out of place and should be deleted. I recommend that the authors make the introduction more concise.*

**Author response**: We agree with this and have incorporated your suggestion to make the introduction more concise*.*

*Methods: The methods section requires a bit more detail.
• Populations and Eligibility: The exclusion criteria are very scanty. For example, were patients with incomplete medical records excluded? If so, this should be included as an exclusion criterion. How did you deal with missing data? Were the retrospective records retrieved from electronic health records, or paper charts, or were there existing stroke registries?*

**Author response**: Thank you for this suggestion. We have changed the exclusion criteria as follows:

“Whereas stroke patients with incomplete medical records about treatment outcome and an unknown date of diagnosis were excluded from the study.” [Pg5, Ln7-8]

We attempted to minimize missing data through thorough data collection and rigorous data validation.

“A complete case analysis of a dataset with missing completely at random was used to deal with missing data.” Methods section, Methods of Data Analysis subsection [Pg7, Ln18-19]

The retrospective records were retrieved from paper charts. We have included it in the methods section, Data Collection Instrument and Procedure subsection:

“A patient's medical records (paper) were used to collect demographic characteristics, outcomes of treatment, comorbidity, medication for treatment, and clinical data such as vital signs and types of strokes.” [Pg6, Ln9-11]

*• What proportion of patients what neuroimaging confirmed strokes? The statement ‘whereas stroke patients whose primary diagnosis was not a stroke (e.g., transient ischemic attacks without stroke confirmation)’ is poorly structured and seems to suggest all patients had neuroimaging. Currently the diagnosis of transient ischemic attacks is tissue based and requires the use of neuroimaging to rule out strokes.*

**Author response**: Thanks for pointing this out. About 77.4% of patients had neuroimaging, and we removed this from the exclusion criteria.

*• The authors also state that ‘transfer-in patients within the follow-up period were excluded from the study’. Considering the hospitals being studied are tertiary facilities, I expect a significant proportion of the patients to be referred from lower-level facilities. Can the authors explain why they were excluded?*

*strokes.*

**Author response**: The reviewer is correct, a significant proportion of patients were expected to be referred, and those were not excluded. The authors intended to state the transfer-in patients. We modified the exclusion criteria.

*• Sampling methods. The authors need to clarify how the sampling frame was constructed and whether any pre-screening was done. How was the list of eligible patients compiled? Were exclusion criteria applied before this apparent random selection of participants?*

**Author response**: Thanks for your comments and suggestions. We have included a flow diagram [Figure 1] and the following sentence:

“A sampling frame was constructed by registering medical record numbers from the logbook of stroke patients.” [Pg5, Ln20-21]

*• Also, it would be helpful to include a flow diagram indicating the total number of records screened. The proportion excluded and reasons for exclusion (the number excluded for incomplete records, transfer-in/ referred from other facilities, etc. and the final number of patients included.*

**Author response**: Done. [Figure1]

*• Regarding the sample size calculation for the second objective, specify assumed proportions or effect size. At least one key factor used.*

**Author response**: Thanks for your comment. We have included the following paragraph in the methods section, Sampling methods subsection:

“The sample size for the second objective (predictors of mortality of stroke patients) was calculated by considering the double population proportion formula by considering different factors that were significantly associated with the outcome variable, like types of strokes (hemorrhagic stroke) with assumed effect size AHR=2.03 (18), Aspiration pneumonia AHR=6.57 (19), Atrial fibrillation AHR=1.104 (20), a two-sided confidence level of 95%, a margin of error of 5%, and a power of 80%, using EPI Info version 7 StatCalc software” [Pg5, Ln12-17]

*• Variables: how was raised ICP? Was it via invasive ICP monitoring? For stroke patients, it is surprising to see heart failure contributing more than other comorbidities like diabetes. This is appropriately highlighted in the discussion section. It would be helpful to indicate how these heart failures were diagnosed in the methods, to clarify the possibility of overestimation. Did the patients have echocardiograms done? Also, why is dyslipidemia not included in the comorbidities?*

**Author response**: This was achieved through non-invasive ICP monitoring. Yes, they had echocardiogram findings. Regarding comorbidities, we took the diagnosis from physician documentation, and those are the common comorbidities that we got from medical record reviews.

*• In retrospective chart reviews, it is impractical to say that "Authors had no access to information that could identify individual participants during or after data collection." This statement should be removed or revised.*

**Author response**: Thank you. It was removed.

*Results:
• The authors state that there was a total of 477 samples which ambiguous. Reword to reflect the fact that a total of 477 records were screened. Also, screen failures should not be included in the total sample size. If patients with incomplete records were excluded, report this both in the methods and in the flow of the results.*

**Author response**: Thank you very much for the comment. The revised text reads as follows:

“A total of 477 records were screened, and 25(5.2%) patients with incomplete information were excluded **(Figure 1).”** [Pg8, Ln17-18]

*• Further classify hemorrhagic stroke into subtypes (e.g., intracerebral hemorrhage, subarachnoid hemorrhage). Provide a more detailed breakdown of stroke-related complications.*

**Author response**: Thank you for this suggestion. It would have been interesting to explore this aspect. However, in the case of our study, stroke was classified into three types: ischemic, hemorrhagic, and unclassified.

“Among 452 patients, about 213 (47.1%) had complications. The most common complications during hospitalization were aspiration pneumonia (20.1%) and ICP/brain edema (20.4%)” [Pg9, Ln14-15]	& Table 2.

*• Do the authors have information regarding the length of stay of patients. Can this be incuded?*

**Author response**: Thanks for your comment. We have included the following:

“The median length of hospital stay was 5 ± 6.0 days (**Table 1**).” [Pg9, Ln1-2]

*• There is no data included on the interventions received by patients such as thrombolysis, thrombectomy, EVD, aneurysmal clipping/ coiling etc. how did the treatments received by patients affect mortality?*

**Author response**: Thank you for pointing this out. Although we agree that this is an important consideration, our study focused on treatments that were being given in the study area, such as antihypertensives, antiplatelets, anticoagulants, Statins, and antibiotics.

• Kindly provide the appropriate labels for the Kaplan-Meier curve to enhance interpretability. The y-axis should be Labelled, and the x-axis should indicate follow-up time or time to event, not “analysis time.” Also, it would be helpful to stratify the curves by important factors such as aspiration pneumonia, etc.

**Author response**: Thank you for this suggestion. We have added the suggested content to the manuscript on [Figure 2 and Figure 3].

“Kaplan Meier failure curve and a log-rank test showed that the differences in cumulative probability of death of patients with hypertension and those without hypertension, with aspiration pneumonia and those without aspiration pneumonia were statistically significant (log‐rank: *p* = 0.0017, log‐rank: *p* = 0.0001, respectively) **(Figure 3).”** [Pg10, Ln5-8]

*Limitations:
• The study only included public hospitals, which could introduce significant bias. This should be included in the limitation.
• 22.6% of cases are unclassified. This is a significant limitation.*

**Author response**: Done.

“The study only included public hospitals, and 22.6% of cases are unclassified, which could introduce a bias.” [Pg15, Ln3-4]

*Response to previous reviewer comments
The authors have failed to adequately address some of the concerns raised by the previous authors. The authors are urged to see the review process as an endeavor to improve the manuscript's quality. Most of the recommendations from the previous reviewers should be incorporated to strengthen the paper.*

**Author response**: Thank you so much. We agree with this and have incorporated your suggestion to make the manuscript stronger.

1. *The revision of the sentence ‘Although infections have received relatively little attention in stroke research’ to ‘Infection is associated with a risk of stroke’ remains out of context. A more accurate phrasing would be: ‘Stroke is associated with an increased risk of post-stroke infections, which can influence outcomes.*

**Author response**: As suggested by the reviewer, we have revised the sentences as:

“Stroke is associated with an increased risk of post-stroke infections (10), and they are a severe consequence for stroke patients in underdeveloped countries with insufficient rehabilitation services (11).” [Pg3, Ln24-26]

2. *As was rightly pointed out by the previous reviewers, the average onset-to-door time of 36 hours is clinically significant and warrants discussion. Although not a primary objective, this is a key finding and should be acknowledged and discussed using insights from relevant regional studies. The delay may reflect systemic issues such as poor road infrastructure, long distances to care, or low public awareness.*

**Author response**: Thank you for pointing this out. The reviewer is correct, and we have discussed it.

“Prehospital care-seeking delays are common among patients with stroke, especially in low-income countries. In this study, the average onset-to-door time is 36 hours. Patients who live farther from hospitals, have a lower level of education, diabetes, hyperlipidemia, or a history of stroke are more likely to experience delays (51). According to a study conducted at Yekatit-12 Hospital Medical College, Ethiopia, age, place of residence, health insurance, and stroke onset time significantly influence the timeliness of seeking medical care (52).” [Pg14, Ln11-16]

Thank you, and we agree that the manuscript is stronger as a result of the suggested

revisions.

Best regards,

Alemayehu Tesfaye (MPH)
